# The Early Fifties. Reminiscences of Guy's Hospital

**Published:** 1909-12-18

**Authors:** 


### SPECIAL ARTICLES.

### THE EARLY FIFTIES.

### REMINISCENCES OF GUY'S HOSPITAL.

AN INTERVIEW WITH MR. THOMAS BRYANT, F.R.C.S.

There are few surgeons who can glance back at so brilliant a record of work as Mr. Thomas Bryant, senior consulting surgeon to Guy's Hospital, Past President of the Royal College of Surgeons of England, President of the Hospitals Association, late President of nearly every medical society in London, and member or corresponding member of many surgical societies in the kingdom and out of it. And there are few leaders so chary of telling you what they have achieved and of dilating on their experiences. You will find "Tom" Bryant, sitting with his back to the light in his little "den," a wonderfully virile and vitalised old gentleman, whose quick, active movements and cheery ringing laugh belie the whiteness of his hair, and make you wonder if there is not some mistake in the directory that puts his qualification as far back as the early 'fifties. If he likes he can tell the story of an interesting life, interesting not merely because every man's life, given in detail, is interesting, but especially so because he has seen, and been instrumental in pushing forward the reforms in hospital and medical work during the last half-century. But he is silent upon the personal. He tells you a wholly absorbing incident, bristling with human interest, and noddingly intimates that he would much prefer if you didn't make use of it. So the best bits go lost, and you must piece together, as best you may, the story of a life that has been so strenuous, and so useful and instructive to everyone who takes an interest in the practical experience of men connected with hospital work.

#### Early Life.

He lived his youth in the Kennington district, where his father, the late Mr. Egerton Bryant, made a large practice and a reputation, finding time, however, in the intervals of his work, to write a monograph on diseases of the larynx, which gained the Fothergillian gold medal of the London Medical Society. Mr. Egerton Bryant was a keen man, and his scientific spirit is evidenced in the fact that he collected and arranged a fine pathological museum, which at his death was handed over to Guy's Medical School, at the request of Sir Astley Cooper, and of which specimens are still to be seen on the shelves of the Gordon Museum. Here at Kennington, in his father's house, young Bryant learned to know some of the leaders of the past. "I remember Sir Astley Cooper," he remarks, speaking of these old times, "visiting us—he was a frequent visitor at my father's—patting me on the back, and asking me what I was going to do. 'Tom must be a doctor,' he remarked to my father, and I believe that first suggestion put the idea into my head. When my father died I was apprenticed to his successor, who was supposed to teach me and another apprentice; but I learned nothing from him, and left him before my time. Finally I entered Guy's and went through my course there, acting as clinical clerk to Dr. Addison and as dresser to Mr. Aston Key, who was a splendid surgeon. In 1849 I qualified, and then—well then my struggles began." They were hard enough struggles, but the recollection of them seems to rouse no sad thoughts, for Mr. Bryant smiles cheerily, perhaps mindful of the fact that he followed the Virgilian advice:

"Ne cede malis, sed semper audentior ito,"

with conspicuous success. His first appointment at Guy's was that of surgical registrar, and he holds that the benefit he derived from that post has been the chief factor in his successful career. To write condensed notes of every case admitted into the surgical wards, to keep the reports up to date, and to discuss with intelligent students the main points in each case for six years—that was informing enough. At least he has found it so; and he impresses on other young teachers the importance of not only being in earnest but of being thorough. Later on, when, as assistant surgeon, he had to attend out-patients twice a week, he adopted a system of teaching which was very helpful and proved highly popular with the students. He divided his cases into classes, and gave to each class a coloured card. There would be a blue card, say, for breast tumours, a black card for rectal cases, and so on. On one day he would take the blue cards, after the urgent cases had been seen, and the students would have the benefit of seeing, say, twenty or more cases of breast tumours in various stages and conditions; on another an equally rich field would be theirs to work at in joint diseases or rectal cases, or some other class. He himself took notes of all these cases systematically, and continued the system in the wards. It is true a certain colleague objected to this system. "He would come into the ward," says Mr. Bryant, "while I was going my round and sneer at me—he called it playing at cards. He would brush past us and call out 'Still playing at cards?' But I didn't mind that, for I saw the scheme was liked by the students, and that it was good teaching and learning for me as well as for them; so I kept it up."

#### His Hospital Work.

When a vacancy occurred on the staff owing to the illness and long absence of Mr. Callaway, Mr. Bryant, who had done Callaway's work for a year, was appointed assistant surgeon. From the first he threw himself whole-heartedly into the scientific part of the work. When he was asked to deliver the Lettsomian lectures he took the subject suggested by the Council of the Society—the Diseases of Children—basing his paper on clinical evidence. The subject, as a special one, was practically untouched, Mr. Holmes's book not having been published, and it attracted so much attention that Mr.

Bryant's volume was subsequently reprinted on the Continent. What he is specially proud of is his connection with the reorganisation of the Cambridge surgical examinations. He was asked by the late Dr. Humphreys, after having been an examiner, to undertake the task of reorganising the surgical examination for the Cambridge B.C., and he did his work so well that few changes have been made since that time. In turn he was called to fill the presidential chair of nearly every medical society in London, with the exception of the Pathological (of which he was Vice-President) and the Obstetrical, finally occupying the highest position as the leader of his profession by being elected President of the Royal College of Surgeons.

With so large a teaching experience it is interesting to hear his observations on modern medical education. There is less thorough clinical teaching than there used to be, he thinks; students depend more on text-book and teacher than on what they themselves observe. His own collection of well-filled note-books, everyone containing sketches, made on the spot, of various interesting lesions, are a proof of this. Few students nowadays have the energy and the patience to write up their work with such minute care. He has always had an outside hobby, and agrees that recreations are necessary for medical men. "They take one out of routine work; they give one a wider interest in life," he remarks, and when you listen to his fishing experiences you feel that he throws himself as whole-heartedly into his play as into his work.

### In Pre-aseptic Days.

The talk veered to old-time hospital work, and he commented upon the changes that have taken place in ward work. "I was dresser to Aston Key—there is his portrait," he remarks, pointing to a fine steel print that hangs on the wall with other mementoes of his hospital life; a keen man's face, sharp and intellectual, with a kindly mouth and level eyebrows. "Yes, he was a fine man," Mr. Bryant observes reflectively. "A grand man; he died of the cholera in 1850. When I was his dresser I well remember we had the last great epidemic of hospital gangrene. I have the picture of some of our cases in mind; it haunts me. Our first case was one of simple fracture of the tibia and fibula with some bruising of the soft parts. When we went round the next day the soft parts round that bruise had become of an ash colour, gangrenous. The whole leg sloughed and had to be amputated, but the poor fellow died. Other cases followed, many being simple contusions and fractures. We dared not operate on any case. One day, towards the end of the epidemic, Key came late. He was a very punctual man, and living as he did at St. Helen's Place, Bishopsgate, he used to stroll up to the hospital. That day we all waited in the colonnade and wondered what on earth had kept him. He came at last and walked straight into the accident ward, and told sister to bring him all the ward sponges. The ward sponges—marine sponges—were brought in an old wooden bowl, and Key took them up one by one and flung each one into the fireplace. 'No more sponges,' he said. 'Whatever are we going to use then?' we asked. 'Whatever you like,' he replied. 'Think it over and tell me next time I come.'" "Well," Mr. Bryant continues, "we did think it over. Of course we had no antiseptics in the strict sense of the word then. We used a little Condy, and there was lots of oakum, coarse, rank-smelling stuff impregnated with tar. Then we used syringes, and finally brushes. I went over to a man in Newcomen Street and ordered five dozen camel-hair brushes. I think I've got one left to show you"—he rummaged in a cupboard and finally brought forward a specimen, an ordinary-looking broad camel-hair brush bound with strong wire. "Key liked these, and we started using them. Each patient had his own brush, which we dipped into iodine solution to clean it, and our results were good on the whole. There was, of course, no real antiseptic surgery in those days. The surgeon operated in an old theatre and in an old operating coat, a frock-coat it was, dirty and stained all over with blood and pus."

### Hospital Cleanliness in the 'Fifties.

"Still you must not think that Guy's was a specially dirty hospital, or that it compared unfavourably with its competitors. By no means. I once took Sir William Ferguson over the hospital—he came one afternoon, and nobody knew that he was there. I took him over the whole place, and he told me when he left that what had impressed him most was our strict cleanliness. I didn't know then that at his hospital they must have been somewhat dirty. Why, would you believe it, under the operating room there was the post-mortem room, separated only by a trap-door in the floor, and Ferguson had been known to do an autopsy below and then come straight up and perform an operation above. Or he would even have a dead body of a case in which he was particularly interested brought up after he had done an operation and was waiting for the next case and would do the post-mortem examination actually on the operating table. No wonder he thought us very clean; for such things, at least, were never done at Guy's, I can assure you. It just shows you how little we knew of antiseptics in those days, though Hunter, to be sure, did know about them. It is true we used iodine water to wash wounds with, and we sealed cases of compound fracture with tinct. benzoinæ co. with some success. Later on iodine water was used as a routine practice, and powdered iodol with boracic acid powder, which I have found very efficient. But now, of course, Lister's method is rightly established and has given us wonderful results."

### Diet and Nursing.

"Other things have changed, of course. Take diet. In my day beer and alcohol were staple articles of hospital diet, and I think we are wrong in a measure to have given them up so entirely. I fully realise that their use has to be restricted, but I also hold that a glass of good beer or porter will not do any harm. Then patients got a cup of tea as a luxury; now tea is provided by the hospital as a matter of routine. We used more medicine than you do now. The nursing then was on the whole uniformly kind, but it was not so good nor half so thorough as it is now. After the 'great nursing row' at Guy's we got a somewhat different class of nurse from those we were accustomed to. I went into my ward one day, after the change, and found one of my patients—a rectal case—prepared for operation in a filthy condition. I called the sister. ' Come and look at this. Whose fault is it? ' I asked. ' Do you expect me to see to that? ' she asked. ' If so I had better go.' ' Well,' I replied, ' if you won't see to it in the future you had much better go, for somebody will have to look after it, and I must never see such a state of things in my ward again.' She was very much flustered, and she didn't like it; but I had no complaint on that score again. That sister became one of my best nurses, and since then all our nurses have fully realised that they have to deal with such cases and that they are there for the patient's sake."

Dealing with modern methods of diagnosis and treatment, Mr. Bryant pointed out how greatly the use of the *x*-rays has influenced and improved modern surgical procedure, but he does not think that the routine use of *x*-rays should be allowed to supersede the ordinary methods of physical diagnosis. "There is really no difficulty in diagnosis if you set about it properly and learn all about the case before you begin to examine it. This is particularly true of cases of fracture or of joint trouble. Some people go the other way about. I remember on a certain day I came into my ward and found one of my dressers at work on a patient's foot, and he was using great force. I asked, ' What are you trying to do? ' He answered, ' I am trying to reduce a dislocated ankle joint.' I looked at the patient's foot, and thought it appeared rather misshapen. On inquiry it proved that it was an old dislocation of many years' standing. Now my friend would have found that out for himself if he had inquired into the history."

A much wider field was opened up when the theme of hospital politics was touched. Mr. Bryant's view as to hospital administration is the conservative one. He has found the voluntary system an ideal one and vigorously supports it. "Don't let us have State interference," he remarks. "I am quite content with the voluntary system, for it is a good system, and it has worked excellently so far. I am opposed to the municipalisation of hospitals; that would be a mistake, I think a great mistake."

